# Misremembrance of Things Past: Depression Is Associated With Difficulties in the Recollection of Both Specific and Categoric Autobiographical Memories

**DOI:** 10.1177/2167702619826967

**Published:** 2019-02-28

**Authors:** Caitlin Hitchcock, Evangeline Rodrigues, Catrin Rees, Siobhan Gormley, Barbara Dritschel, Tim Dalgleish

**Affiliations:** 1 Medical Research Council Cognition and Brain Sciences Unit, University of Cambridge; 2 Cambridgeshire and Peterborough National Health Service Foundation Trust; 3 School of Psychology and Neuroscience, University of St Andrews

**Keywords:** assessment and intervention, autobiographical memory, depression, open data, open materials

## Abstract

Impaired retrieval of specific, autobiographical memories of personally experienced events is characteristic of major depressive disorder (MDD). However, findings in subclinical samples suggest that the reduced specificity phenomenon may reflect a broader impairment in the deliberate retrieval of all autobiographical memory types. This experiment (*N* = 68) explored this possibility by requiring individuals with and without MDD to complete a cued-recall task that required retrieval of specific, single-incident memories to a block of cues; retrieval of categoric, general memories to a block of cues; and to alternate between retrieval of specific and general memories for a block of cues. Results demonstrated that relative to never-depressed controls, individuals with MDD experience reduced recall of both specific (*d* = 0.48) and general memories (*d* = 1.00) along with reduced flexibility in alternating between specific and general memories (*d* = 0.90). Findings support further development of autobiographical memory–based interventions that target a range of retrieval deficits rather than specificity alone.

Autobiographical memory plays a fundamental role in daily cognition. We typically draw on autobiographical memory hundreds of times a day to facilitate problem solving (Jing, Madore, & Schacter, 2016), imagine and make plans for our future (Jing et al., 2017), and facilitate shared relationship discourse (Beike, Brandon, & Cole, 2016). Disruption to autobiographical memory retrieval therefore, understandably, has a detrimental effect on daily functioning. Retrieval of an autobiographical memory requires successful navigation within a complex, multilevel autobiographical memory store. Models of autobiographical memory propose that autobiographical information is stored hierarchically, with categoric generalizations that summarize similar experiences (e.g., going to school) accessible at the top of the hierarchy and information regarding contextual detail of specific, single events (e.g., my final year history exam) stored at the bottom of the hierarchy (Conway & Pleydell-Pearce, 2000). This allows memories to be retrieved at different levels of granularity from general summaries to more detailed single-event memories, and both of these memory types are important in daily functioning. Generalized memories form the basis from which we make judgments about ourselves and the world (Klein, Cosmides, Tooby, & Chance, 2002) and provide a heuristic for planning future events (Williams et al., 2007). Specific memories help us to cognitively reappraise difficult situations, solve problems, and populate the details of future plans by providing detailed information about what has worked in the past (Jing et al., 2016) and set boundary conditions for the validity of generalized memories (Hitchcock, Rees, & Dalgleish, 2017).

There is consistent evidence that targeted retrieval of autobiographical memories is impaired in mental health problems such as depression. In particular, there is prolific evidence that depressed individuals experience difficulties when trying to recall specific memories. A widely used evaluation of an individual’s profile of autobiographical recollection is the Autobiographical Memory Test (AMT)—a series of cue words of negative, positive, or neutral valence to which participants are asked to recollect specific personal memories and in which the dependent variable of interest is the relative proportion of specific (vs. general) memories successfully retrieved (Williams & Broadbent, 1986). On the AMT, depressed individuals consistently retrieve a lower number of specific memories than healthy controls (Dalgleish et al., 2007; Williams et al., 2007). Importantly, this reduced specificity does not appear to be simply an epiphenomenon of the depressed state but rather independently predicts depressive prognosis (Sumner, Mineka, & Griffith, 2010), purportedly through reducing the aforementioned daily cognitive skills that rely on recall of specific memories (e.g., cognitive reappraisal, problem solving). Numerous literature reviews have now established reduced memory specificity as a cognitive characteristic of depression (Hitchcock, Nixon, & Weber, 2014; Sumner, Griffith, & Mineka, 2010; van Vreeswijk & de Wilde, 2004; Williams et al., 2007). Consequently, memory specificity has been proposed to represent a unique intervention point for shifting depressive prognosis (Craske, 2018; Dalgleish & Werner-Seidler, 2014).

A number of treatment protocols have emerged that seek to improve memory specificity, and thereby depression, in accordance with recommendations to translate cognitive science into novel, precision-based intervention approaches (cf. National Institute for Mental Health’s Research Domain Criteria; Insel et al., 2010), and a recent meta-analysis highlighted the promise of such approaches (Hitchcock, Werner-Seidler, Blackwell, & Dalgleish, 2017). The most widely evaluated of these is Memory Specificity Training (Raes, Williams, & Hermans, 2009), although several other protocols have been developed (e.g., Life Review Therapy; Korte, Bohlmeijer, Cappeliez, Smit, & Westerhof, 2012; Serrano et al., 2012). In light of evidence that memory specificity protocols produce significant treatment effects that are comparable in size to other evidence-based interventions (Hitchcock, Werner-Seidler, et al., 2017; Werner-Seidler et al., 2018), memory specificity interventions are increasingly being evaluated in samples diagnosed with other psychiatric conditions, including posttraumatic stress (Moradi et al., 2014), panic (Korrelboom, Peeters, Blom, & Huijbrechts, 2014), and eating disorders (Korrelboom, de Jong, Huijbrechts, & Daansen, 2009).

A tendency to navigate away from specific detail and toward generalizations about the self and the past is also a key feature of the theoretical framework underlying cognitive therapy for depression, which proposes that overgeneralization of negative events is a core mechanism underlying the disorder (Beck, 1967, 2008). Reduced specificity in memory retrieval therefore fits nicely with extant evidence-based models of treatment. However, there is evidence from analogue studies to suggest that the phenomenon is perhaps broader than simply difficulties with memory specificity and may in fact reflect a more fundamental impairment in the ability to successfully navigate the autobiographical memory store to intentionally retrieve any type of personal memory. Dalgleish et al. (2007) demonstrated that subclinical symptoms of depression were associated not only with reduced recall of specific memories on the AMT but also with a reduced ability to recall generalized, categoric memories when explicitly instructed to do so on a Reversed Instructions version of the AMT (the AMT-R). Building on this work, Dritschel, Beltsos, and McClintock (2014) sought to assess flexibility in autobiographical retrieval using an Alternating Instructions version of the AMT (AMT-AI) that combines the standard AMT with the AMT-R and additionally requires individuals to alternate between retrieval of specific and general memories. Dritschel et al. found that reduction in the ability to alternate between retrieval of specific and general memories was associated with higher subclinical symptoms of depression. These analogue findings in individuals with subclinical low mood suggest that clinical depression may not be simply characterized by reduced memory specificity but potentially also with reduced ability to deliberately retrieve general memories and flexibly move between retrieval of different autobiographical memory types.

Given the strong ongoing focus on poor memory specificity and the resources being invested in developing and evaluating memory specificity interventions, it is critical to determine that the mechanism being targeted is the most valid representation of the underlying difficulty. An overly circumscribed definition of the mechanism of change is likely to compromise the potential efficacy of any mechanism-driven, process-focused intervention. In this study, we therefore sought to determine whether the difficulties with the flexible retrieval of autobiographical memories (Dritschel et al., 2014) and categoric memory retrieval (Dalgleish et al., 2007) found in those with subclinical levels of low mood critically also characterize clinical depression.

In evaluating differences in autobiographical retrieval between groups of participants—in this case, a clinically depressed group and a never-depressed healthy control group—it is critical to ensure that any evidence of impaired deliberate retrieval is not simply due to group differences in broader aspects of executive control deficit. It is therefore essential to match groups on an executive function task that is known to correlate with AMT performance independent of depressive status. One such measure is verbal fluency (see Dalgleish et al., 2007), and we therefore included a fluency measure to ensure that any group differences in autobiographical retrieval were not systematically influenced by any group-based differences in executive capacity.

Our specific hypotheses were that on the AMT-AI (Dritschel et al., 2014), individuals with a diagnosis of major depressive disorder (MDD), currently in episode, relative to never-depressed control participants, would demonstrate a broad deficit in the targeted retrieval of both specific (Williams et al., 2007) and categoric memories (Dalgleish et al., 2007) when presented in separate blocks and also when mixed in an alternating block. We further hypothesized that there would be an added retrieval cost for depressed individuals when asked to flexibly switch between specific and categoric recall in the alternating block relative to either recall type alone in the separate blocks (as in Dritschel et al., 2014).

## Method

### Participants

On the basis of the moderate effect size for the relationship (*d* = 0.60, directional α = .05) between AMT-AI performance and depressive symptoms observed by Dritschel et al. (2014), data were collected from 34 healthy community volunteers with no previous history of psychiatric disturbance who were registered on our department’s panel of volunteers (control group) and 34 (depressed group) individuals with a diagnosis of MDD experiencing a current Major Depressive Episode (MDE). The depressed group was also invited to participate in a subsequent clinical trial of an autobiographical memory–based intervention reported elsewhere (Hitchcock, Gormley, et al., 2018). All consented to participate in the trial. These depressed individuals were recruited from our department’s panel of volunteers with a history of depression. Diagnostic status was determined by trained research staff using the Structured Clinical Interview for *DSM* Disorders (SCID; First, Williams, Karg, & Spitzer, 2015), under supervision of a clinical psychologist who second-rated each SCID. Discrepancies were resolved via discussion, and this resulted in 100% agreement on diagnostic status for primary and comorbid disorders. Both panels of volunteers comprise individuals who have responded to print and online advertisements requesting volunteers to participate in research at the MRC Cognition and Brain Sciences Unit.

For both groups, exclusion criteria were intellectual disability, traumatic brain injury, or current substance/alcohol use disorder. For healthy control participants, exclusion criteria also comprised presence of a current or prior diagnosis of a *DSM* disorder and/or score of 13 or more (above the cutoff for the mild range) on the Beck Depression Inventory II (BDI-II; Beck, Steer, & Brown, 1996). Two potential control participants were excluded on this basis. Groups were matched on age, gender, and highest level of received education (see Results).

### Materials

#### AMT-AI (Dritschel et al., 2014)

The AMT-AI is an adaption of the original AMT (Williams & Broadbent, 1986). The AMT measures the ability to deliberately retrieve specific memories (i.e., “memory of one specific time or one specific event”) in response to a series of cue words of positive, negative, or neutral emotional valence. The AMT-AI extends the original AMT by requiring individuals to recall specific autobiographical memories to a series of six cue words, recall categoric autobiographical memories (i.e., “memory of an event that has happened on many different occasions”) to a series of six cue words (as required in the AMT-R; Dalgleish et al., 2007), and alternate between recall of specific and categoric memories for 12 cue words. The order of these specific (AMT-S), categoric (AMT-R), and alternating (AMT-A) blocks was randomized between participants. Two lists of cue words were randomized between participants—the original list used by Dritschel et al. (2014) and a second list we created to match the number of positive (*n* = 8), negative (*n* = 8), and neutral words (*n* = 8) and cue frequency in the English language (Wilson, 1988), *F* < 1. All cue words were taken from the MRC Psycholinguistic Database (Wilson, 1988) and randomized between blocks. Before completing test trials, participants were given four practice trials (two each for specific and categoric memories), and feedback was provided in response to incorrect answers.

Task instructions were presented on a computer, and following an instruction to recall either a specific or categoric memory, participants were given 1 min to press a computer key to indicate they had a memory in mind. Participants then reported their memory aloud. Responses were audio-recorded and later coded as to whether they were specific, categoric, extended (i.e., event lasting longer than one day), or repeated (i.e., a memory that had been previously reported) memories; a semantic associate (i.e., information related to the cue which is not a memory); or an omission (i.e., could not think of a memory). In accordance with prior literature, responses that were reported after 30 s had passed (indexed as the computer-recorded number of seconds between cue presentation and the key press) were scored as omissions. Ten percent of audio recordings were coded by a second rater. There was good (Cicchetti, 1994) interrater reliability—intraclass correlation coefficient = .75. Because of the uneven number of trials between blocks, we used proportions correct in each block as our dependent variable. The proportion of correct responses was calculated as the number of memories recalled in line with the instructions for that block divided by the number of trials minus the number of omissions, as per Dritschel et al. (2014). Results remained the same when the number of omissions was not subtracted.

#### Executive control

We administered measures of executive control to ensure that groups were comparable on verbal executive abilities pertinent to AMT-AI performance. The Verbal Fluency Task (VFT; Spreen & Strauss, 1998) assesses executive control over verbal information and was included because it is an established measure of executive processes involved in AMT performance (Dalgleish et al., 2007). Participants were given 60 s to generate words in a given category (animals, foods, or occupations) and a further 60 s to generate words beginning with a certain letter (*F, A, S*). We recorded the number of correctly identified words in each condition (incorrect responses are repeated words or proper nouns or words that did not fit the category/letter). The Digit Span task from the Wechsler Adult Intelligence Scale (Wechsler, 2010) was also administered to index working memory and ensure that our assessment of executive control did not rely on any single task (Royall et al., 2002).

#### Symptom measure

The BDI-II consists of 21 items that assess depressive symptoms and severity over the past 2 weeks. The scale is valid and reliable. A score of 13 or below is within the normal/nonclinical range, 14 to 19 reflects the mild range, 17 to 29 reflects the moderate range, and 30 and above reflects the severe range of symptom severity (Beck et al., 1996).

### Procedure

Ethical approval was obtained from the NHS National Research Ethics Committee (11/H0305/1). After providing written informed consent, participants individually completed the AMT-AI, VFT, Digit Span, and BDI-II in a quiet testing room on a single occasion. All depressed participants had previously completed the SCID to assess MDD diagnosis and comorbidity, and both depressed and control participants completed the Mood Module of the SCID (to index history of depression and diagnostic status) during the testing session. Assessment sessions lasted 45 min to 60 min, and participants were reimbursed at a rate of £6 per hour for their time, plus travel expenses.

## Results

### Sample characteristics

Descriptive statistics are presented in Table 1. The depressed and control groups were comparable on age, *t*(66) = 0.14, *p* = .89, *d* = 0.04; gender, χ^2^(3) = 2.68, *p* = .44; and level of education, Fisher’s exact test = 3.44, *p* = .53. Importantly, the groups were also closely matched on levels of executive ability as indexed by scores on the Digit Span Test, *t*(66) = 1.21, *p* = .23, *d* = 0.29, and verbal fluency, *t*(66) = 0.15, *p* = .88, *d* = 0.17. Groups differed on depressive symptoms in the anticipated direction, *t*(38.82) = 12.69, *p* < .001, *d* = 3.08. The mean BDI-II score for the depressed group was in the severe range (Beck et al., 1996). The mean number of previous depressive episodes was 3.53 (*SD* = 1.74), with nine of the depressed participants having experienced too many episodes to count the distinct number, as coded on the SCID. One depressed participant met criteria for diagnosis of current obsessive compulsive disorder, eight met criteria for current generalized anxiety disorder, and two met criteria for current posttraumatic stress disorder. Lifetime diagnoses included panic disorder (*n* = 1), posttraumatic stress disorder (*n* = 1), eating disorder (*n* = 1), alcohol/substance abuse (*n* = 3), and social anxiety disorder (*n* = 1). Thirty-nine percent of depressed participants were currently taking antidepressant medication, and 19% were receiving psychological treatment.

**Table 1.** Mean Sample Characteristics by Group

Characteristic	Depressed (*n* = 34)	Controls (*n* = 34)
Age	33.97 (13.27)	33.50 (13.58)
Number of females	20	18
White participants (%)	70.6	76.5
Education level	1; 11; 2; 12; 8	0; 7; 1; 13; 13
Verbal Fluency Task	19.96 (5.09)	19.12 (4.84)
Digit Span	18.65 (4.48)	19.94 (4.31)
BDI-II	29.50 (11.50)	3.38 (3.43)

Note: Values in parentheses are standard deviations. BDI-II = Beck Depression Inventory–Second Edition; Education level = number completed Year 11; sixth form; diploma/additional training; undergraduate degree; postgraduate degree (UK system).

### AMT-AI performance

**Fig. 1.**
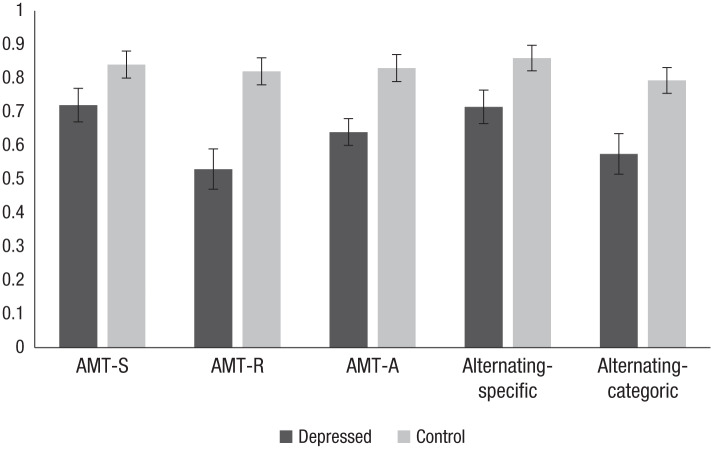
Mean proportions of memories correctly recalled in the specific (AMT-S), reversed (AMT-R), and alternating (AMT-A) blocks and for specific trials (alternating-specific) and categoric trials (alternating-categoric) in the alternating block of the Autobiographical Memory Test–Alternating Instructions. Error bars indicate standard error.

Accuracy in the three individual blocks of the AMT-AI did not vary as a function of the order in which the blocks were presented, *Fs* ≤ 1, *p*s > .39. A multivariate analysis of variance examining the proportion of correct responses across the three conditions (AMT-S, AMT-A, AMT-R; see Fig. 1) with group as a between-subjects factor demonstrated a significant multivariate effect of group, Wilks’s lambda = 0.75, *F*(3, 64) = 6.98, *p* < .001. The planned follow-on univariate analyses revealed that in line with our hypotheses, the depressed group demonstrated a lower proportion of correct responses than controls for the AMT-S, *F*(1, 66) = 3.90, *p* = .05, *d* = 0.48 95% confidence interval (CI) = −0.02, 0.98]; AMT-R, *F*(1, 66) = 17.05, *p* < .001, *d* = 1.00, 95% CI = [0.48, 1.52]; and AMT-A blocks, *F*(1, 66) = 13.82, *p* < .001, *d* = 0.90, 95% CI = [0.38, 1.42]. Results remained the same when covarying for verbal fluency and working memory measures to further account for executive function, Wilks’s lambda = 0.76, *F*(3, 62) = 6.57, *p* = .001. Our hypotheses were therefore supported.^1^

To evaluate whether there was a performance cost of retrieving memories in the alternating condition relative to the single memory type blocks (cf. Dritschel et al., 2014), we compared the proportions of specific/categoric memories correctly recalled in the AMT-S/AMT-R blocks against the proportion of those memories recalled in the AMT-A block (see Fig. 1). We completed two mixed analyses of variance (ANOVAs) (for specific and categoric memories, separately) with block (single, alternating) as the within-subjects factor and group as a between-subjects factor. Again, significant effects of group revealed that across block types, depressed participants demonstrated fewer correct responses than controls—specific memories, *F*(1, 66) = 6.45, *p* = .01, *d* = 0.62 [95% CI, 0.12, 1.12], categoric memories, *F*(1, 66) = 19.81, *p* < .001, *d* = 1.08, 95% CI = [0.55, 1.61]—but there was no significant effect of block or any Block × Group interaction for either memory type, *F*s < 1, and all effect sizes were trivial (*d*s < 0.2). Again, effects remained the same when working memory and verbal fluency measures were covaried, *Fs* < 1, *d*s < 0.2. There was therefore no support for an additional cost of alternating instructions on recall of either specific or categoric memories.

## Discussion

The current findings demonstrated that relative to never-depressed control participants, individuals with a diagnosis of MDD experienced difficulties with the intentional recollection of both specific and categoric autobiographical memories, although there was no support for an additional performance cost in depression when participants had to alternate from trial to trial between one memory type and another. Interestingly, larger effect sizes relative to controls were observed for deliberate recall of general memories (*d* = 1.00) and the ability to alternate between specific and general memories (*d* = 0.90) compared with the deliberate recall of specific memories (*d* = 0.48) in those with depression. These findings are consistent with the notion that reduced memory specificity, as consistently observed in depressed samples (Williams et al., 2007), is in fact only one component of a more fundamental deficit in the ability to intentionally retrieve autobiographical memories of different types. This has implications for the conceptualization of the autobiographical memory difficulties driving depressive symptom change and the consequent translational development of emerging science-driven interventions.

There are a number of factors that may reduce the ability to successfully navigate the autobiographical memory store and correctly retrieve a predefined memory type, as elucidated in the CaRFAX model (Williams et al., 2007) proposed to explain the established memory specificity difficulty in depression. These include goal neglect during the retrieval process and the retrieval search becoming hijacked by either the internal affective context in which retrieval occurs (Hitchcock, Golden, Werner-Seidler, Kuyken, & Dalgleish, 2018) or self-relevant information that is activated during the search. Each of these factors would abort the memory retrieval process before the predefined search criteria were filled whether the search was for a specific memory or a categoric memory.

Although further research is needed to explore the wider cognitive mechanisms impairing directed retrieval (for review, see Sumner, 2012), the current pattern of results is unlikely to be simply a function of more domain-general executive performance difficulties associated with depression because our depressed and comparison samples were matched on an executive measure associated with memory retrieval independent of depressive history, and results remained significant even when performance on this measure was covaried.

There are some potential limitations of the present study that merit discussion. The AMT-AI did not contain sufficient trials of different valence to enable us to evaluate any differential performance to positive and negative cues. Given the prolific negative bias in memory recall observed in depression, further examination of valence effects is warranted. We also only assessed cued recall of specific and categoric memories, replicating prior work with subclinical samples (Dalgleish et al., 2007; Dritschel et al., 2014), but we anticipate that deliberate retrieval of extended memories is also likely to be impaired in depression, and this idea is in need of future examination. We failed to replicate the analogue finding of an association between depressed mood and additional difficulties in alternating between specific and categorical memories (Dritschel et al., 2014). However, the original finding was a correlation between self-reported symptoms on the BDI and AMT-AI performance in a student sample. The fact that our study was powered for a case-control design meant that we were unable to examine the replicability of these symptom-severity effects directly once the absence of any group differences became clear. Finally, inclusion of a task-switching measure to supplement our two indices of executive control also would have been ideal. However, given that we found no support for any additional difficulties in alternating between specific and categorical recollection in depression on the AMT-AI, it is unlikely that unmeasured group differences in task switching are confounding our results.

This study evaluates for the first-time performance on the AMT-R (Dalgleish et al., 2007) and AMT-AI (Dritschel et al., 2014) in participants with clinical depression. Successful navigation of autobiographical memory appears important in supporting a number of cognitive processes that are central to daily life. The generalized summaries provided by categoric memories guide efficient decision making (Cosmides & Tooby, 2000; Klein et al., 2001), whereas specific memories play an important role in problem solving (Jing et al., 2016) and facilitating social interaction (Beike et al., 2016)—everyday skills that are compromised during depression, subsequently driving functional impairment. Further, we recently demonstrated that interaction between generalizations and specific memories may serve to shape emotionally valenced self-evaluations (Hitchcock, Rees, et al., 2017). Improving the ease with which depressed individuals can generate these different memory types on demand and move between them may therefore help alleviate symptoms of depression. Similarly, ameliorating memory retrieval difficulties may aid the efficacy of cognitive behavioral therapy (CBT). Improved ability to access general memories, particularly of a positive nature, may support the strengthening of positive generalizations about the self (e.g., “I am worthy”)—a key goal of CBT. Further, improved ability to move between memory types may aid CBT tasks that require an individual to move between general and specific levels of information (e.g., planning behavioral experiments).

Current autobiographical memory–based interventions have focused on improving recall of specific memories, but our findings suggest that explicitly training improved recall of all memory types may mitigate more appropriately the autobiographical retrieval issues experienced by the clinically depressed. Indeed, there is evidence that intervention to improve the flexibility of memory retrieval may have a positive impact on symptoms of depression (e.g., Hitchcock et al., 2016; Hitchcock, Gormley, et al., 2018), and the current results support further development of such interventions. Although current evidence suggests that improving specificity of recall is likely to yield beneficial results for depressive symptoms, the current findings suggest that intervention efficacy may be improved by targeting deliberate retrieval of all memory types.

## Supplemental Material

Hitchcock_OpenPracticesDisclosure – Supplemental material for Misremembrance of Things Past: Depression Is Associated With Difficulties in the Recollection of Both Specific and Categoric Autobiographical Memories Click here for additional data file. Supplemental material, Hitchcock_OpenPracticesDisclosure for Misremembrance of Things Past: Depression Is Associated With Difficulties in the Recollection of Both Specific and Categoric Autobiographical Memories by Caitlin Hitchcock, Evangeline Rodrigues, Catrin Rees, Siobhan Gormley, Barbara Dritschel and Tim Dalgleish in Clinical Psychological Science

## Supplemental Material

Hitchcock_Supplemental_Figure – Supplemental material for Misremembrance of Things Past: Depression Is Associated With Difficulties in the Recollection of Both Specific and Categoric Autobiographical Memories Click here for additional data file. Supplemental material, Hitchcock_Supplemental_Figure for Misremembrance of Things Past: Depression Is Associated With Difficulties in the Recollection of Both Specific and Categoric Autobiographical Memories by Caitlin Hitchcock, Evangeline Rodrigues, Catrin Rees, Siobhan Gormley, Barbara Dritschel and Tim Dalgleish in Clinical Psychological Science

## Notes

1. We completed a post hoc analysis to investigate whether there was any differential effect between groups for specific versus categorical recall. A mixed ANOVA with block (AMT-S, AMT-R) as the within-subjects factor and group as the between-subjects factor revealed the expected main effect of group, *F*(1, 66) = 12.40, *p* = .001, *d* = 0.85, 95% CI = [0.34, 1.36], a significant effect of Block, *F*(1, 66) = 10.55, *p* = .002, *d* = 0.79, 95% CI = [0.28, 1.30], and a significant Group × Block interaction, *F*(1, 66) = 5.78, *p* = .02, *d* = 0.58, 95% CI = [0.08, 1.08]. Paired *t* tests revealed that performance in the control group was not significantly different across blocks, *t*(33) = 0.63, *p* = .54, but interestingly that the depressed group performed significantly worse at retrieving categoric memories than specific memories, *t*(33) = 3.83, *p* = .001.
