# Supplementary material for: Misremembrance of Things Past: Depression Is Associated With Difficulties in the Recollection of Both Specific and Categoric Autobiographical Memories
Source: Clin Psychol Sci. 2019 Feb 28;7(4):693–700. doi: 10.1177/2167702619826967 (PMC7324083; doi:10.1177/2167702619826967)
Supplement: Hitchcock_Supplemental_Figure – Supplemental material for Misremembrance of Things Past: Depression Is Associated With Difficulties in the Recollection of Both Specific and Categoric Autobiographical Memories [file Hitchcock_Supplemental_Figure.pdf]

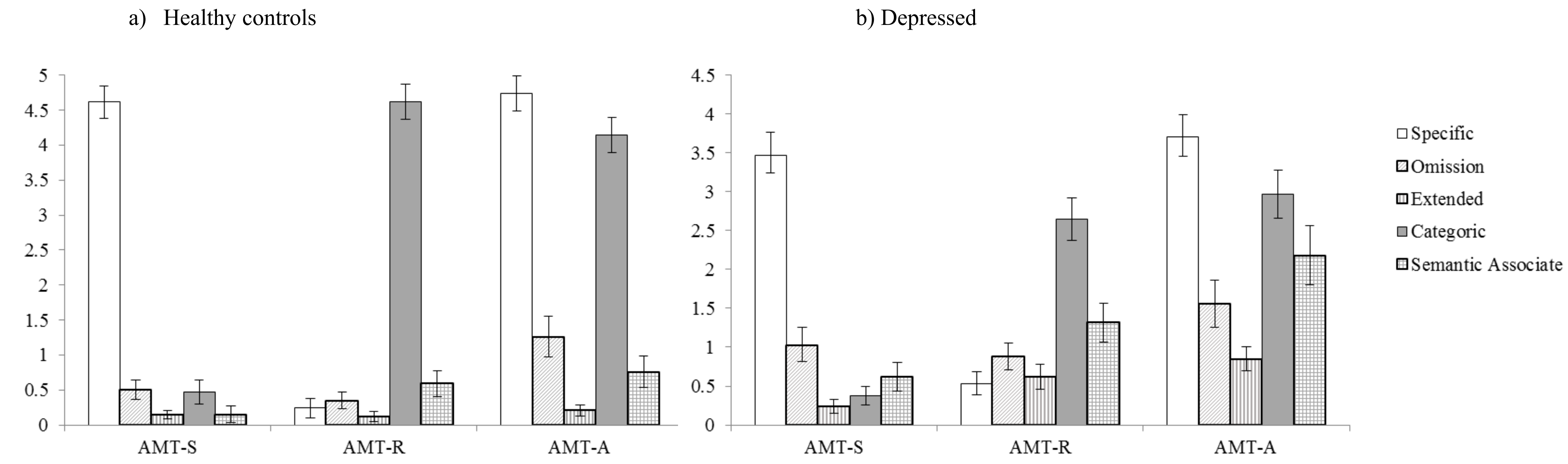

Supplementary Figure 1. Number of each memory type recalled in the AMT-S, AMT-R, and AMT-A blocks of the Alternating Instructions Autobiographical Memory Task, by Group.
